# Investigating and characterizing the binding activity of the immobilized calmodulin to calmodulin-dependent protein kinase I binding domain with atomic force microscopy

**DOI:** 10.1186/s13065-017-0360-7

**Published:** 2017-12-06

**Authors:** Xiaoning Zhang, Hongmei Hu

**Affiliations:** 1 College of Biotechnology, Southwest University, Chongqing, 400715 China; 2 Key Laboratory of Mariculture and Enhancement of Zhejiang Province, Marine Fishery Institute of Zhejiang Province, Zhoushan, 316021 China

**Keywords:** Protein–protein interactions, Calmodulin, CaM kinase I binding domain, Atomic force microscopy, Micro/nanometer-scale

## Abstract

Protein–protein interactions are responsible for many biological processes, and the study of how proteins undergo a conformational change induced by other proteins in the immobilized state can help us to understand a protein’s function and behavior, empower the current knowledge on molecular etiology of disease, as well as the discovery of putative protein targets of therapeutic interest. In this study, a bottom-up approach was utilized to fabricate micro/nanometer-scale protein patterns. One cysteine mutated calmodulin (CaM), as a model protein, was immobilized on thiol-terminated pattern surfaces. Atomic Force Microscopy (AFM) was then employed as a tool to investigate the interactions between CaM and CaM kinase I binding domain, and show that the immobilized CaM retains its activity to interact with its target protein. Our work demonstrate the potential of employing AFM to the research and assay works evolving surface-based protein–protein interactions biosensors, bioelectronics or drug screening.

**Electronic supplementary material:**

The online version of this article (10.1186/s13065-017-0360-7) contains supplementary material, which is available to authorized users.

## Introduction

Protein patterning techniques in micro/nanometer-scale has demonstrated its huge potentials in bio-sensing and bio-analysis field [1–3]. The main advantages of these protein micro/nano-arrays technologies include high detection sensitivity, low consumptions of reagent samples (nL level), and a few protein requirements [4]. Typically, upon binding of ligand to the immobilized protein, there is a change in protein conformation. This ligand-mediated conformation change can be devised to alter the scientific signal of biosensor, which can be analyzed by assessing any of its observable properties (e.g. optical or electrochemical properties).

Calcium, like many other inorganic elements, plays key roles in a variety of biological processes, such as the blood-clotting process, metabolism and signal transduction. Lots of Ca^2+^ dependent proteins exist in the cytoplasma of cells, calmodulin (CaM) is one of them, which is ubiquitous in almost all eukaryotic cells [5]. CaM is a small (148 amino acid residues), acidic (PI = 4.3), and heat-stable protein, which can be exposed to temperatures higher than 90 °C and remains stable. Calcium-bound CaM (Ca^2+^/CaM) can bind and activate a series of kinases in order to mediate the effects of Ca^2+^ [6–8]. The multifunctional Ca^2+^/CaM-dependent protein kinase I, also known as CaM kinase I (CaM KI) is a well-known effector of calcium- and CaM-mediated functions. It is found in many tissues, but in neurons it has especially high concentrations, and it may be up to 2% of the total protein in some brain regions. Based on Dzhura’s work, the CaM KI mediates phosphorylation and plays a fundamental part in triggering I_ca_ facilitation, which responses to the intracellular Ca^2+^ concentration [9, 10]. When an external stimulus increases intracellular Ca^2+^ levels, it increases the amount of Ca^2+^/CaM. Ca^2+^/CaM then bind to the autoinhibitory domain of the CaM KI *α*-subunit and activate CaM KI by causing the binding domain to dissociate from the autoinhibitory domain. The activated CaM KI migrates to the post-synaptic density (PSD), phosphorylates α-amino-3-hydroxy-5-methyl-4-isoxazolepropionic acid receptors (AMPA receptors), which are ionotropic transmembrane receptors, and enhances their activity to decrease the Ca^2+^ level. Therefore, CaM activates CaM KI by displacement of its binding domain, and the capability of CaM to bind with CaM KI binding domain is able to indicate the activity of CaM to interact with CaM KI.

Protein–protein interactions, which are responsible for many biological processes [11, 12], have been extensively studied through a number of alternative ways, such as fluorescence technique [13, 14], electrophoresis [15, 16], microcalorimetry [17], et al. However, most of those techniques characterize protein–protein interaction in bulk solution. Only a small percentage of the published work done to reveal how proteins undergo a conformational change induced by protein–protein interaction in the immobilized state, and AFM is one of techniques used. Besides, AFM could help us to understand the architecture of a protein and a multiprotein complex in air directly. In addition, AFM is the only microscopic technique which is capable of visualizing biomolecules at the single-molecule level with sub-nanometer accuracy. Because AFM allows studying the adhesion, elasticity, association process, dynamics and other properties of biological sample, it is able to help us to quantitatively analyse protein–protein interactions to reveal the nature and magnitude of forces and the related binding energy landscape. For example, by attaching one of the interacting proteins to the AFM tip and the other protein to the sample surface, the molecular binding forces can be quantified from the positive binding/rupture events [18].

In the present work, a protein immobilization protocol is used for the controlled and oriented immobilization of Ca^2+^/CaM. AFM was utilized to evaluate this procedure and investigate the interaction between the immobilized Ca^2+^/CaM and the CaM KI binding domain. Ca^2+^/CaM and CaM KI binding domain were concerned as subjects in the case of this study because their interactions in bulk solution have been fully studied by circular dichroism (CD), nuclear magnetic resonance (NMR), and electron paramagnetic resonance (EPR) [19]. The structure of CaM KI and the substrate sequence recognition motif for CaM KI are therefore clear.

## Experimental

### Chemicals and materials

#### Chemicals for surface preparation

Octadecyltrichlorosilane (OTS, 97%) and (11-mercaptoundecyl)trimethoxysilane (MUTMS, 95%) were purchased from Gelest. Toluene (HPLC grade) was purchased from Fisher Scientific. Ultraflat silicon (100) wafers (N-type) were purchased from Sigma-Aldrich Corporation. Sulfuric acid and hydrogen peroxide were purchased from Sigma-Aldrich Corporation.

#### Materials for CaM expression, purification, and reaction

Luria–Bertani (LB) broth, used to grow the cell culture, and Tris(2-carboxyethyl) phosphine hydrochloride (TCEP) disulfide reducing agent were purchased from Sigma-Aldrich Corporation. Calcium chloride (CaCl_2_) was purchased from Flinn Scientific. CaM was purified using chitin beads from New England Biolabs. 2-anilinonaphthalene-6-sulfonic acid (ANS) used for fluorescence experiment and SDS-PAGE were obtained from Invitrogen Corporation. Calmodulin—dependent protein kinase I (299–320) binding domain, which is a putative CaM-binding region, was obtained from AnaSpec. All the solution was prepared with water from a Millipore Direct-Q UV water purification system.

### Protein expression and purification

Purification and expression of genetically engineered CaM with cysteine on N-terminus is based on instructional manual prepared by New England Biolabs [20]. In order to prevent dimer formation, TCEP was applied in protein solution. SDS-PAGE was used to confirm the CaM purity (see Additional file 1).

In our experiment, we used 2,6-anilinonaphthalene sulfonate (ANS) fluorescent probe to test the bio-activity of the purified solution-state Ca^2+^/CaM. It is well established that solvent-exposed hydrophobic surfaces are formed upon Ca^2+^ binding to CaM, and ANS binds to the hydrophobic parts of proteins through polar interactions and can be monitored by the increase in fluorescence emission intensity, which demonstrates the activity of Ca^2+^/CaM indirectly [21]. When EDTA is added to the solution, Ca^2+^ is removed from Ca^2+^/CaM, and the hydrophobic binding pocket disappears. This conformational change causes the release of bound ANS from CaM to the aqueous solutions, leading to a decrease in fluorescence intensity. Therefore, by monitoring the fluorescence intensity variation we can confirm the conformational change in CaM, which is an indication of CaM viability [22].

**Fig. 1:**
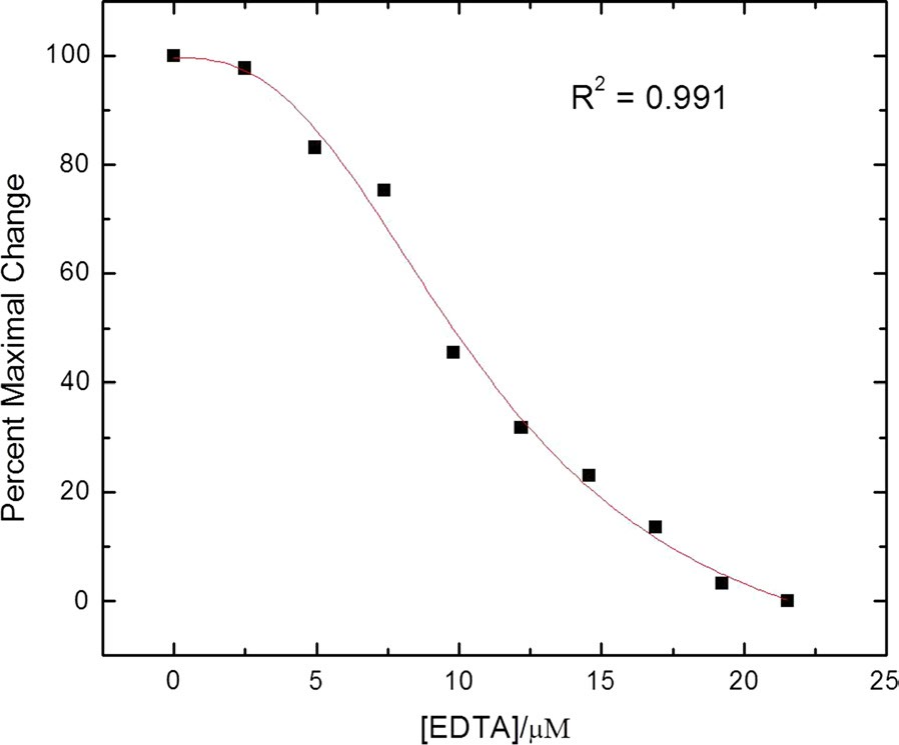
EDTA titrations of ANS labeled CaM monitored by ANS fluorescence emission measurement. For purpose of comparison, all the fluorescence intensities were normalized to their respective 100% change. Sigmoidal fitting along with coefficient of determination (*R* ^2^) were also demonstrated in Fig. 1

During the experiment, the protein was labeled with a 1:1 ratio of ANS overnight at room temperature followed by dialysis against the same buffer. 1 µL increments 0.5 mmol L^−1^ EDTA was added into the 400 µL CaM solution each time. The solution was excited at 310 nm, and emission spectra in the range from 400 to 500 nm were obtained with a Perkin Elmer LS-55 fluorescence spectrometer. Figure 1 shows a sigmoidal shape of the binding curve which was observed by adding EDTA solution into CaM solution accumulatively. As expected, the increase of EDTA amount led to a decrease in fluorescence signal intensity due to the release of ANS caused by EDTA-induced CaM conformational change. The fluorescence intensity change indicates that our purified CaM was capable of changing its conformation properly in the solution state.

### Surface fabrication

The fabrication and characterization of the chemical pattern were performed with an Agilent PicoPlus 3000 AFM in an environmental chamber. AFM can provide atomic-level resolution in *z* axis. The Si (100) wafer was cut into 1 cm × 1 cm pieces. Then, the wafer was boiled in the piranha solution (two parts of 98% sulfuric acid and one part of 30% hydrogen peroxide) at 170 °C for 30 min. At high temperature, the H_2_O_2_ was decomposed; O· and OH· were generated to remove all organic contaminants and also help to grow a thin oxide layer of silanol (Si–OH) on the surface. After that, the wafer was dipped into 5 mmol L^−1^ OTS toluene solution for a pinhole-free OTS-coated wafer fabrication, which was capable of being used for the follow-up experiment [23–26].

**Fig. 2:**
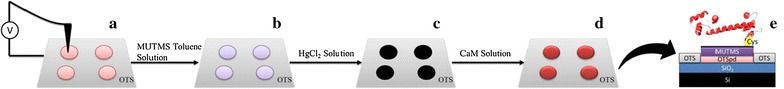
The Scheme for CaM patterns fabrication. **a** The OTSpd disk patterns were fabricated by local oxidation lithography. **b** MUTMS was cross-linked onto the OTSpd patterns, converting the OTSpd patterns into thiol-terminated surfaces. **c** Substrate was then incubated into HgCl_2_ solution to form Hg-SH coupling. **d** Cysteine-mutated CaM was immobilized on the chemical patterns via cysteine-Hg-SH coupling. **e** Structural model of substrate corresponding to part (**d**)

The experimental scheme was shown in Fig. 2. Chemical patterns on the OTS coated Si wafer were fabricated using local oxidation lithography first (Fig. 2a). With the help of the chemical patterns, we are able to modify surface with defined chemistry and create topography with references in positions and height. A detailed description of the OTS partially degraded pattern (OTSpd) fabrication has been demonstrated in Additional file 1, and an OTSpd pattern fabrication set-up was demonstrated in Additional file 1: Figure S2 [27].

From the AFM topography histogram (Additional file 1: Figure S3b), we can know the depth of the OTSpd pattern is 10.60 ± 0.01 Å lower than the OTS background. The depth of the OTSpd chemical pattern provides a height reference for calculating the thickness of other parallel layer on itself. Although some studies applied AFM cross-section profile to analyze the height of object [28–30], it is believed that AFM topography histogram can better represent the average height change of pattern areas in the present work due to the protein film, which is immobilized on the chemical patterns, exhibiting an “unflat” surface. Histograms of the corresponding heights were fitted to two Gaussian functions by using MicroCal Origin software in order to enable a quantitative comparison. The distance between these two peaks is the height of the disk pattern [31].

**Fig. 3:**
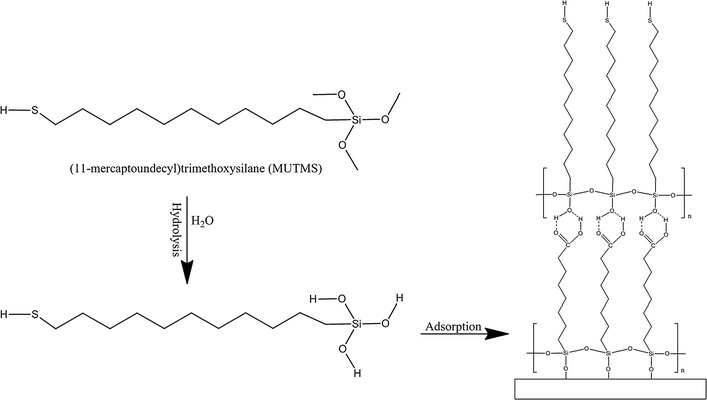
Schematic representation of the construction of a MUTMS monolayer on the OTSpd surface

After the OTSpd patterns were fabricated, the substrate was rinsed in 10% hydrochloric acid for 10 min and cleaned with the super-critical carbon dioxide snow jet cleaner from Applied Surface Technologies. The possible electrostatic charges and contaminates were completely removed as a result of above procedures. Then, the pattern was soaked in a 10 mmol L^−1^ MUTMS toluene solution overnight to convert the carboxylic acid-terminated OTSpd surface pattern to a thiol-terminated surface pattern (Fig. 2b). The structure and formation of MUTMS layer on OTSpd pattern is illustrated in Fig. 3. MUTMS molecules react with the trace amount of water in the solution, forming silanols in the first step. Then the silanols cross-linked and selectively anchored on the hydrophilic OTSpd surface. The pattern in Additional file 1: Figure S4 is a representative MUTMS silane monolayer self-assembled on top of the OTSpd pattern. From AFM characterization, the height of the MUTMS pattern over the OTS background is 10.62 ± 0.02 Å.

Then, the sample with MUTMS patterns was incubated into 10 mmol L^−1^ HgCl_2_ solution for half an hour to form SH-Hg coupling, as shown in Fig. 3c, which will be used to immobilize cysteine-mutated CaM. 5 μg mL^−1^ CaM with buffer solution (25 mmol L^−1^ Tris–HCl, 1 mmol L^−1^ CaCl_2_, pH 8.0) was deposited onto the pattern area for one hour in refrigerator at 4 °C (Fig. 3d) [32]. Then the sample surface was wiped with a piece of ChemWipe paper, in a typical force of 1 N [33], to remove the nonspecifically adsorbed protein on the OTS background, while those specifically bind to substrate surface remained.

### Surface characterization

Because AFM imaging in liquid environment provides a less accurate measurement [34], and it is difficult to interpret the AFM phase image taken in liquid environment [35]; CaM patterns were imaged at 75% relative humidity environment (at 25 °C) in air in ac mode with MikroMasch NSC-14 tips. The imaging set point was maintained at 99% of the tip free oscillation amplitude so that the tip tapped the CaM immobilized surface under a minimal force. Because the tip touched the protein surface in the humid environment, a possible electrostatic charge from the sample was dissipated after the tip touched the sample. Hence, the height measurement was not affected by the protein’s electrostatic charge. All AFM images were processed using WSxM [36].

## Results and discussion

**Fig. 4:**
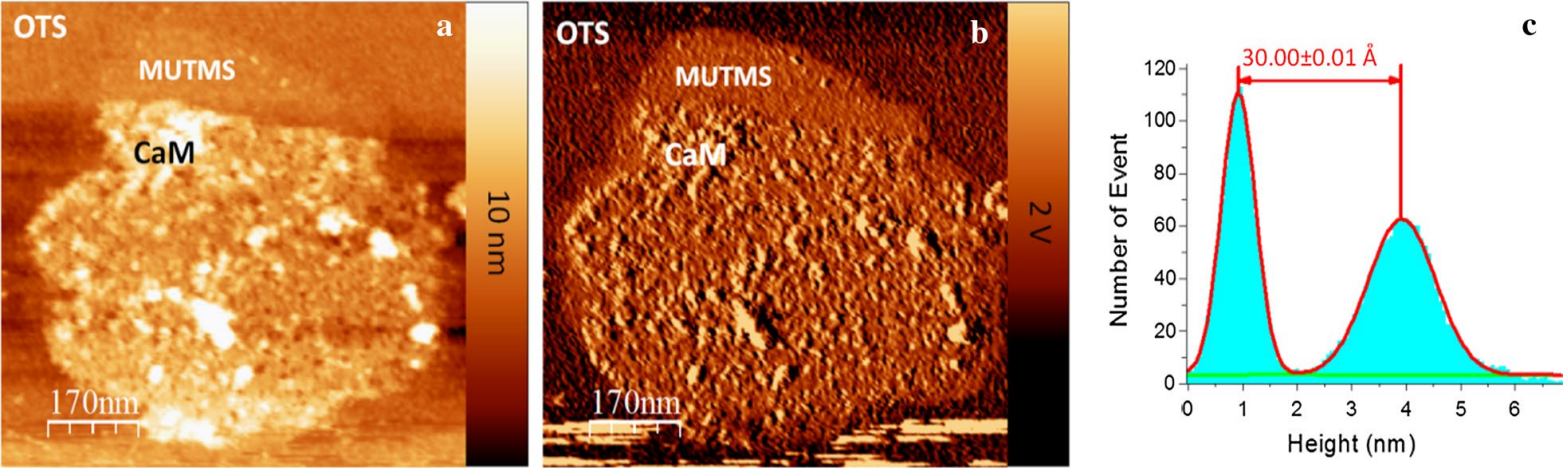
A partially covered CaM layer on the MUTMS pattern. **a** Ac mode topography image. **b** Corresponding phase image. **c** Histogram corresponding to protein fully covered area in (**a**). The distance between the two peaks in the histogram specifies the height of the CaM pattern over OTS background in (**a**)

The MUTMS modified surface was used to immobilize cysteine-mutated CaM through cysteine-Hg-SH coupling. Figure 4a demonstrates a protein pattern in which protein film was made only partially covered the MUTMS disk intentionally. Therefore, Fig. 4a includes the surface features of OTS, MUTMS, and protein. To create protein molecules partially covered patterns, we swabbed the surface with a piece of ChemWipe paper in a force greater than 5 N. Under such condition, ChemWipe paper can remove protein molecules that are non-specifically adsorbed on the OTS background, and also scratch off some protein molecules which are specifically immobilized on the chemical template. AFM topography characterizations show that after protein immobilization procedure, the height of the patterns changed to 3.00 ± 0.01 nm above the OTS background (Fig. 4a). In the corresponding phase image (Fig. 4b), the phase signal of the MUTMS pattern area is 282.18 ± 68.34 mV, which is different from the phase signal of protein pattern area 122.67 ± 88.2 mV, indicating they have different surface identities [37]. From both AFM topography and phase signal, we can conclude that CaM was immobilized on the MUTMS chemical pattern.

**Fig. 5:**
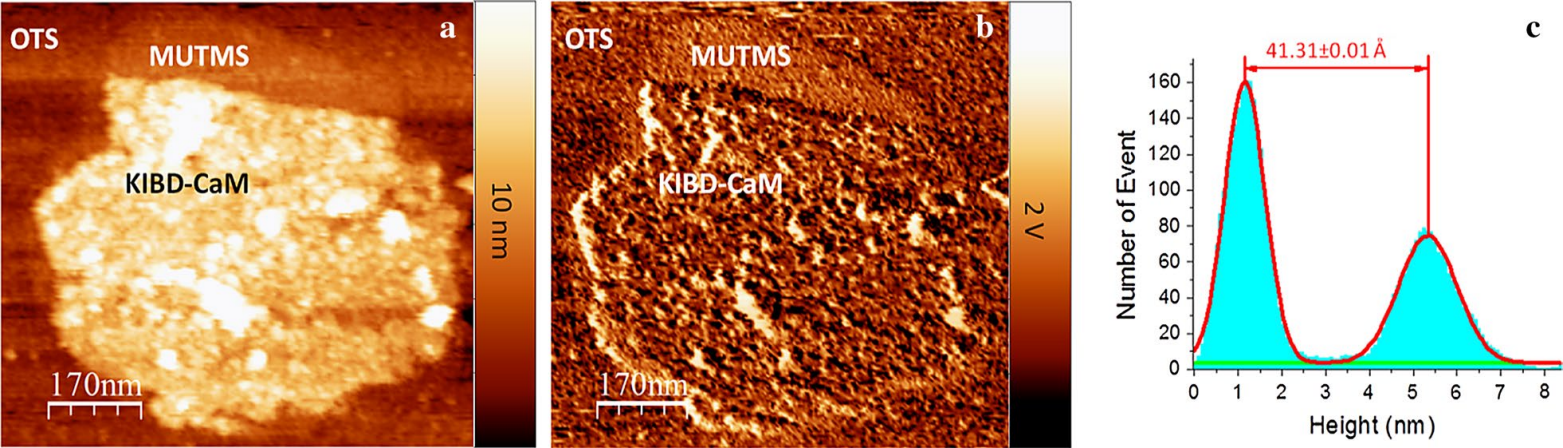
Sample in Fig. 4 was incubated in CaM KI binding domain solution for 10 min. **a** AFM ac mode topography image. **b** Corresponding phase image. **c** Histogram corresponding to protein fully covered area in (**a**). The distance between the two peaks in the histogram specifies the height of the KIBD-CaM pattern over OTS background in (**a**)

CaM KI binding domain is an amino acids 299 to 320 fragment of the CaM KI, which can independently bind CaM and be utilized for CaM interaction studies [38]. Ca^2+^/CaM can capture this fragment by wrapping tightly around it, inducing a calmodulin conformational change. In the experiment, the immobilized CaM was soaked for 10 min in a 1 g mL^−1^ CaM KI binding domain solution at 4 °C. Figure 5a, b show the CaM pattern, after treatment with CaM KI binding domain solution for 10 min and then rinsed with copious amounts of buffer solution, in topography and phase channels, respectively. The MUTMS/OTS border, protein/MUTMS border, and protein/OTS border are recognizable in the phase image indicating the surface was not covered by CaM KI binding domain. The clean, protein uncovered MUTMS surface (Fig. 5a) indicates the non-specifically adsorbed protein molecules were removed. AFM tip was manipulated to scan on the surface of protein pattern multiple times. The height of the protein pattern maintained the same after the AFM tip scanning, indicating that the interaction between CaM KI binding domain and the immobilized CaM is specific. Otherwise, the non-specifically adsorbed CaM KI binding domain could be wiped off by AMF tip during its scanning on surface, and the height of the protein pattern should decrease correspondingly. The results from AFM histogram (Fig. 5c) reveals that the CaM KI binding domain causes the height of the CaM layer to increase 11.31 ± 0.10 Å, which indicates that the immobilized CaM still remained activity to bind its target protein.

**Fig. 6:**
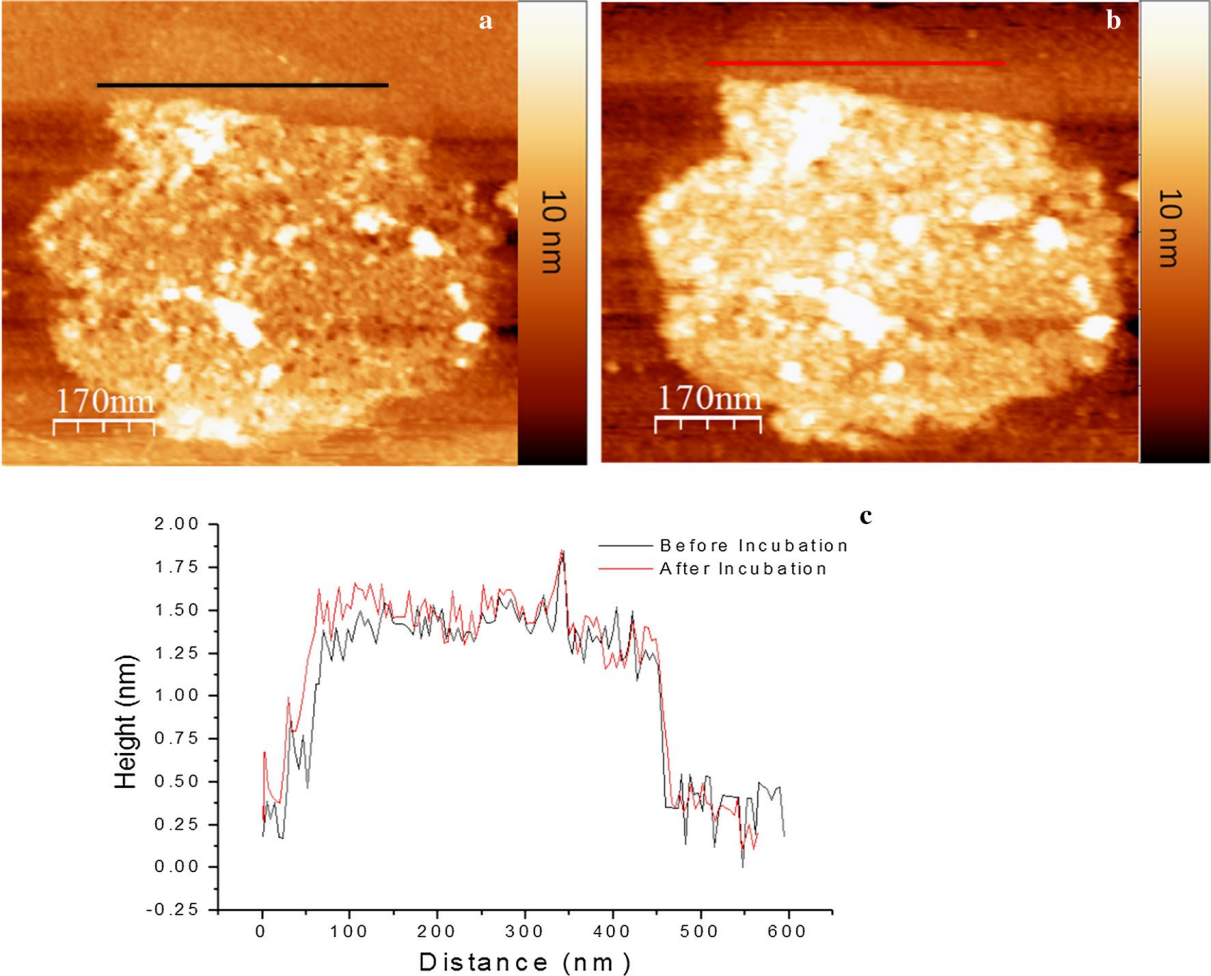
CaM KI binding domain can bind immobilized CaM (**a**) inducing a conformational change (**b**). The height cross-sectional profiles of the same position on MUTMS patterned area in (**a**) and (**b**) were plotted in (**c**)

In Fig. 6, we plot the height cross-sectional profiles corresponding to the same location of MUTMS pattern before (black line) and after (red line) the CaM KI binding domain solution incubation. Cross-sectional profiles (Fig. 6c) show that the height of MUTMS above OTS background remains the same after the CaM KI binding domain solution incubation, indicating no CaM KI binding domain bound on the MUTMS surface.

MUTMS, CaM, and CaM KI binding domain-bound CaM (KIBD-CaM) patterns were also characterized for different samples to obtain better statistical results. The final results are summarized in Table 1.

**Table 1:** Height of the surface patterns

	Apparent height above OTS (nm)	N
MUTMS	1.08 ± 0.18	30
CaM	2.95 ± 0.06	18
KIBD-CaM	4.20 ± 0.09	15

## Conclusions

Our results show that the immobilized CaM retains its activity to interact with its target protein. Upon conformation change to KIBD-CaM, the apparent height of the CaM molecules increased. Our results demonstrate the feasibility of employing AFM to probe and understand the protein–protein interaction. We expect to find wide applications of this present methodology in surface-based protein–protein interactions biosensors, bioelectronics or drug screening.

## Additional file

**Additional file 1.** Supporting information.

## Abbreviations

CaM: calmodulin
AFM: atomic force microscopy
OTS: octadecyltrichlorosilane
OTSpd: OTS partially degraded
MUTMS: (11-mercaptoundecyl)trimethoxysilane
LB: Luria–Bertani
TCEP: Tris(2-carboxyethyl)phosphine hydrochloride
ANS: 2-anilinonaphthalene-6-sulfonic acid
CaM KI: CaM kinase I/CaM-dependent protein kinase I
PSD: post-synaptic density
AMPA receptors: phosphrylates á-amino-3-hydroxy-5-methyl-4-isoxazolepropionic acid receptors
KIBD-CaM: CaM KI binding domain-bound CaM
