# Supplementary material for: Investigating and characterizing the binding activity of the immobilized calmodulin to calmodulin-dependent protein kinase I binding domain with atomic force microscopy
Source: Chem Cent J. 2017 Dec 6;11:128. doi: 10.1186/s13065-017-0360-7 (PMC5718999; doi:10.1186/s13065-017-0360-7)
Supplement: Supplementary file 1 — Additional file 1. Supporting information. [file 13065_2017_360_MOESM1_ESM.docx]

**Supplementary Material**

**Calmodulin for SDS-PAGE**

In the experiment, CaM purity was confirmed by SDS–PAGE analysis. Figure S1 shows photography of SDS-PAGE gel. The right side lane is the MW standard (Prestained Protein Molecular Weight Marker, Thermo Scientific; from top to bottom bands are 120, 85, 50, 35, 25 and 20 kD), while the left side lane was loaded with purified CaM. Previous studies demonstrated that molecular weight of CaM is around 17 kDa (1). Our SDS-PAGE analysis indicated that only one 17 kDa-band was observed, which suggested that CaM was successfully purified.

**Figure S1.** SDS-PAGE analysis indicated that only one 17 kDa-band was observed.

**AFM Local Oxidation Lithography**

In the experiment, a Mikromasch Type CSC17 platinum−titanium-coated conductive AFM tip was used in the pattern fabrication process and was connected to the virtual ground. During fabrication, a 5 to 10 V DC bias was applied to the OTS-coated wafer. In this case, the Pt-Ti-coated tip acts as the cathode and the OTS wafer acts as the anode. Since the voltage applied is higher than the water electrolysis reaction needs (1.23 V), the water between the tip and the sample is electrolyzed, generating active oxygen species such as ozone, atomic oxygen, and hydroxyl radical, which can oxidize the methyl-terminated OTS film to carboxylic-terminated patterns(2). This partially degraded OTS pattern is called an OTSpd pattern. Figure S2 shows a representative OTSpd pattern fabricated set-up using a stationary tip under the above controlled conditions.


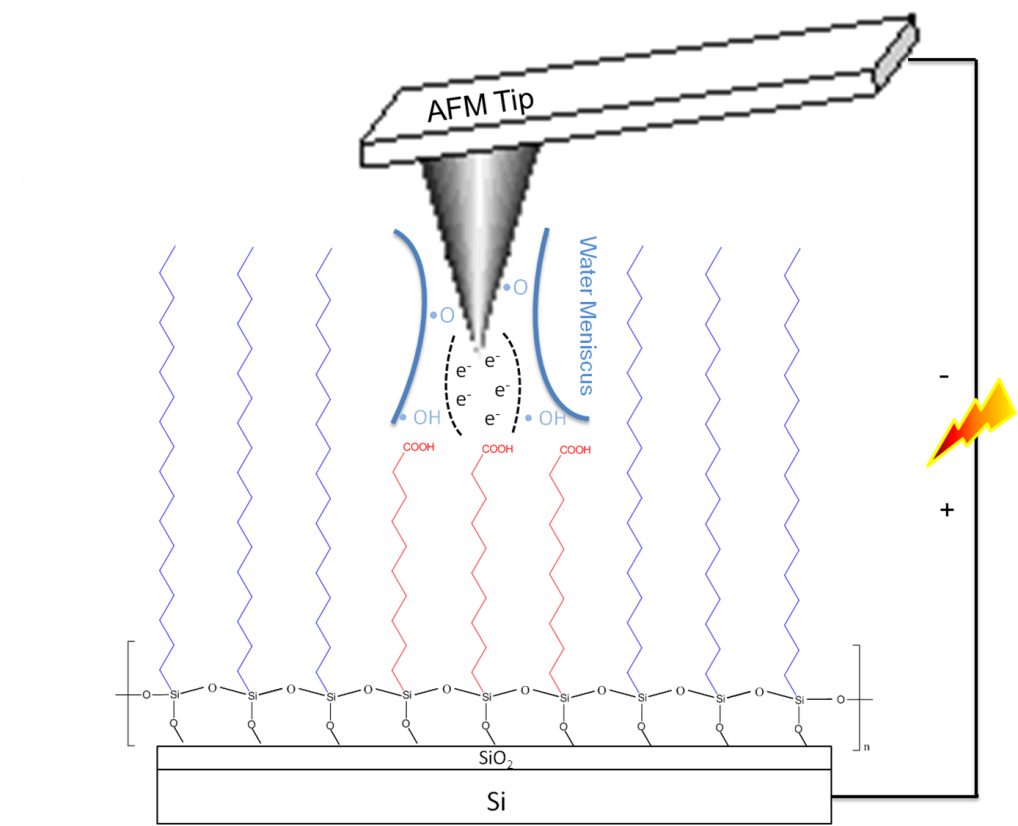


**Figure S2.** The experimental setup for the AFM probe local oxidation lithography on OTS film.

The AFM topography histogram in Figure S3b demonstrates the height distribution of the OTS background and the OTSpd pattern, which shows the visual impression of the distribution of the height of each pixel. The topography histogram is an estimation of the probability distribution of a continuous variable. The peak-fitting program in MicroCal Origin was used to fit the height histogram into two Gaussian-shaped peaks. The distance between these two fitted peaks is the depth of the OTSpd pattern.


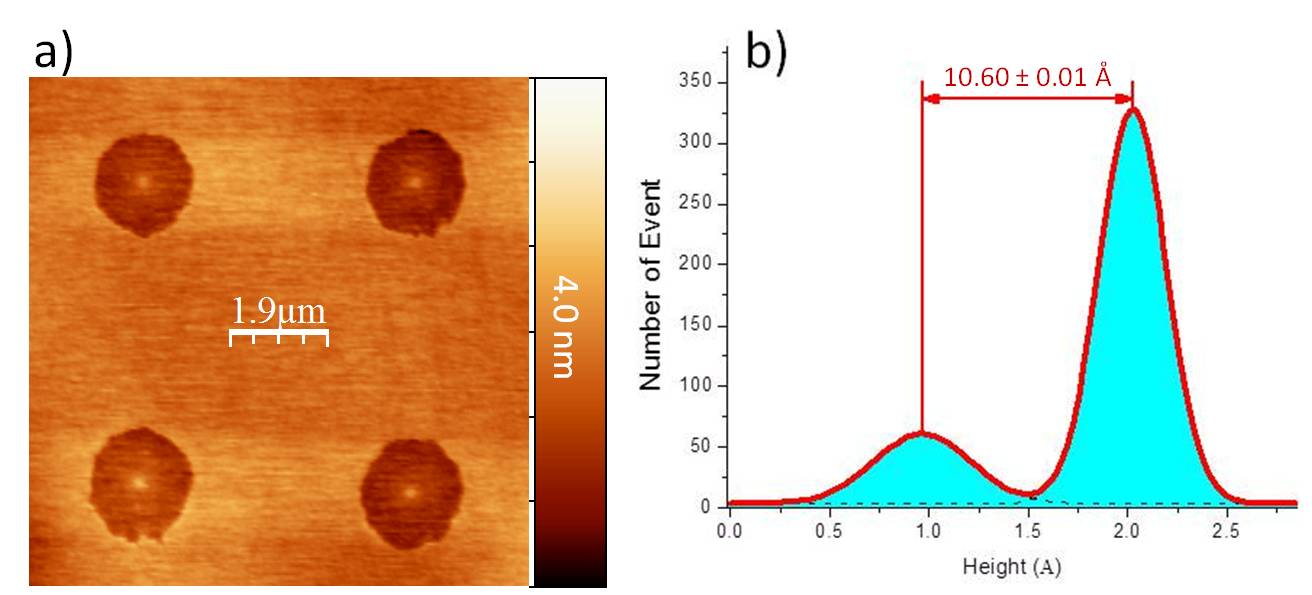


**Figure S3.** Measuring the depth of OTSpd pattern. (a) The AFM topography image of an array of OTSpd patterns, acquired in contact mode immediately after the sample was prepared. (b) The histogram of the topography image, which indicates the depth of the OTSpd disk is 10.60±0.01 Å lower than the OTS background.

**Thiol-terminated Surface Fabrication**

The pattern in Figure S4a is a representative MUTMS silane monolayer self-assembled on top of the OTSpd pattern. Figure S4b is the corresponding height histogram. The distance between these two peaks is the height of the MUTMS pattern over the OTS background, which is 10.62±0.02 Å. Since the OTSpd chemical template is 10.60±0.01 Å lower than the OTS background, we derive that the MUTMS silane layer has a height of 21.22±0.02 Å.


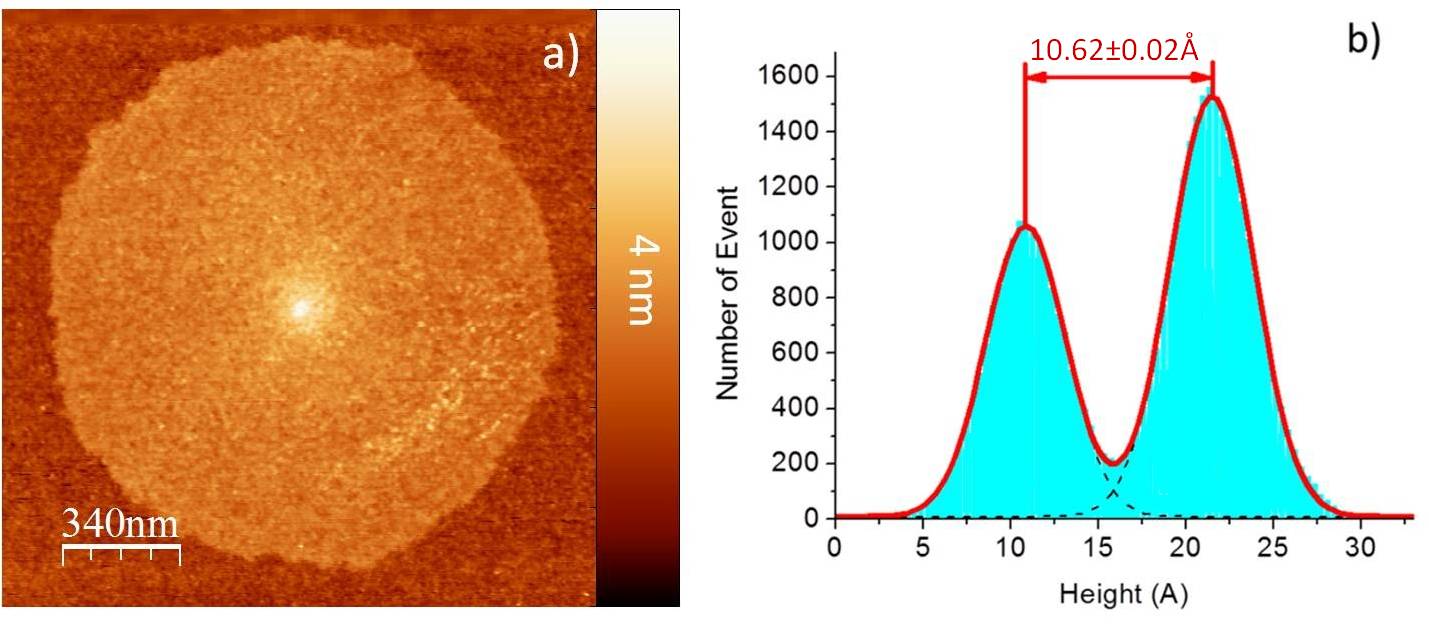


**Figure S4.** Panel (a) is an AFM topography image of the MUTMS pattern. Panel (b) is the histogram corresponding to panel (a). The distance between the two peaks in the histogram specifies the height of the MUTMS pattern in Figure S4a.

**References**

1. Gomes AV, Barnes JA, Vogel HJ. Spectroscopic Characterization of the Interaction between Calmodulin-Dependent Protein Kinase I and Calmodulin. Archives of Biochemistry and Biophysics. 2000;379(1):28-36.

2. Cai Y. The partially degraded hydrophilic silane pattern and its application in studying the structures of long chain alkane films. Langmuir the Acs Journal of Surfaces & Colloids. 2009;25(10):5594-601.
